# A Novel Deletion Mutation of the F8 Gene for Hemophilia A

**DOI:** 10.3390/diagnostics12112876

**Published:** 2022-11-21

**Authors:** Jingwei Wang, Jian Gu, Hongbing Chen, Qian Wu, Liang Xiong, Bin Qiao, Yan Zhang, Hongjun Xiao, Yongqing Tong

**Affiliations:** 1 Department of Clinical Laboratory, Renmin Hospital of Wuhan University, Wuhan 430060, China; 2 Clinical Molecular Diagnosis Institute, Wuhan University, Wuhan 430060, China; 3 Department of Pulmonary and Critical Care Medicine of Renmin Hospital, Wuhan University, Wuhan 430060, China; 4 Medical Vocational and Technical School, Wuhan University, Wuhan 430060, China

**Keywords:** hemophilia A, coagulation factor FVIII, gene mutation, bleeding disorder

## Abstract

**Background:** Hemophilia A (HA) is an X-linked recessive blood coagulation disorder caused by a variety of abnormalities in F8 gene, resulting in the absence of impaired molecule production of factor VIII (FVIII) in the plasma. The genetic testing of the F8 gene encoding FVIII is used for confirmation of HA diagnosis, which significantly reduced serious complications of this disease and, ultimately, increased life expectancy. **Methods:** Sanger sequencing was performed in F8 gene exons of the suspected patients with blood coagulation-related indicators. **Results:** A novel F8 indel variant c.6343delC, p.Leu2115SerfsTer28 in exon 22 of the F8 gene was identified in the suspected families. The infant with this novel variant appeared the symptom of minor bleeding and oral cavity bleeding, and decreased activity of FVIII, which is consistent with that of F8 deleterious variants. The 3’D protein structural analysis of the novel variant shows a change in FVIII protein stability, which may be responsible for the pathogenesis of HA. **Conclusions:** A novel deleterious variant was identified in our case, which expands the F8 variants spectrum. Our result is helpful for HA diagnosis and benefits carrier detection and prenatal diagnosis. Our study also reveals that mutation screening of the F8 gene should be necessary for HA suspected patients.

## 1. Introduction

Hemophilia A (HA, OMIM: 306700), is an inherited X-linked recessive bleeding disease. The clinical manifestations of this disease include prolonged clotting time and repeated bleeding since childhood. Severe hemophilia A can be life-threatening. Mutations in F8 gene are the genetic basis of HA [1]. The individuals with F8 gene mutations have impaired FVIII function in the plasma, which causes bleeding tendency, and the bleeding severities are correlated with the plasma FVIII activity. HA predominantly affects males, and around 1/3 of newly diagnosed HA patients have no family history, but genetic defects occur spontaneously or sporadically [2,3]. 

HA is diagnosed based on the bleeding history and abnormal coagulation tests in the patients and families. The abnormalities in the coagulation test include prolonged activated partial thromboplastin time (APTT) and decreased FVIII activity. HA is classified into three phenotypes according to FVIII activity levels in plasma: severe (<1% of normal activity), moderate (1–5%), and mild (5~40%). Severe phenotype occupies most of the cases (60%), moderate in 15%, and mild in 25% of all the cases [4]. However, only approximately of 30% of HA heterozygous females have FVIII activity lower than 40%, which is unreliable for diagnosis [5]. 

F8 gene (ID: 2157) encoding the coagulation factor VIII locates on the X chromosome (Xq28), and consists of 26 exons and 25 introns. The F8 precursor polypeptide is composed of 2351 amino acids, it forms the mature protein containing 2332 amino acids by removing 19 signal peptides from N-terminal. The mature F8 polypeptide includes six domains organized in the order A1-A2-B-A3-C1-C2 (from N to C terminus), in which A1 and A2 domains form a heavy chain; A3, C1, and C2 domains are light chain; and B domain is a connecting region. Different domains have specific functions. The C2 domain determines the stability and the activity of FVIII by binding to von Willebrand factor (VWF) and phospholipids (PLs [6]. The A2 domain and A1/A3-C1-C2 dimer contribute to FIXa binding [7].

The molecular diagnostics not only benefit for HA diagnosis confirmation, but also for female carrier’s identification, prenatal diagnosis, inhibitor risk development prediction, disease biology and clinical phenotype definition, etc. In this study, the genetic defect of F8 gene are screened by PCR-sanger sequencing method in HA patients, and one novel mutation in F8 gene was identified in a male newborn with severe HA.

## 2. Materials and Methods

### 2.1. Patients

**Figure 1:**
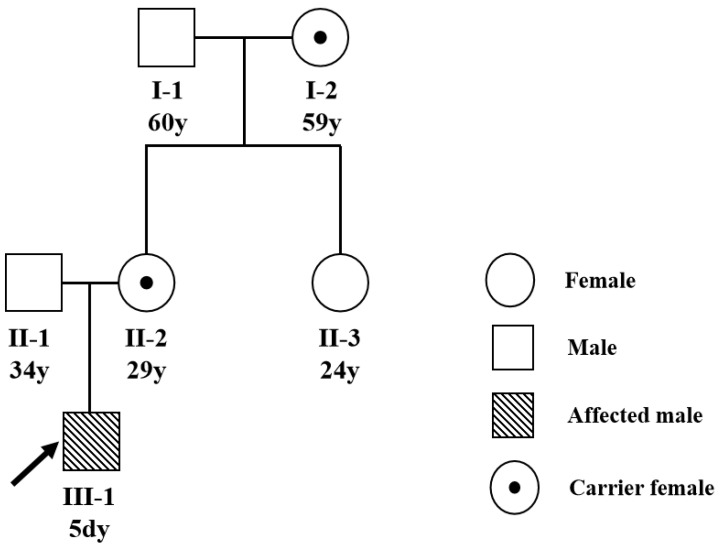
A pedigree for the hemophilia A family. The three-generation family pedigree of the proband was drawn. The inheritance patterns of the family were X-linked, as indicated by the familial pedigrees. Squares and circles indicate males and females, respectively. Darkened Squares represent the affected male members. The black dotted circles represent female carrier. Proband are denoted by the slanted arrow.

A 5-day-old male was admitted to our hospital with abnormal coagulation function and infant jaundice (yellowing of a baby’s skin and eyes) after a vaginal delivery, with a serum bilirubin level >14 mg/dL. The baby had a normal birth weight (under 4.16 kg), was combination feeding. A fusiform (2.5 cm ×1.5 cm) scalp hematoma could be seen in the left temporal part, the right elbow joint was swollen and thickened, the surrounding skin was bruised, and the movement as not obviously restricted. Soft tissue ultrasound indicated an anechoic area (5.1 × 1.6 × 1.1 cm) around the right biceps brachii (considering the hematoma). The family has no history of marriage between close relatives, and a three-generation pedigree was drawn (Figure 1).

### 2.2. Clinical Characteristic and Laboratory Test

Blood samples were collected in the patients and their family members with the informed consent. The complete blood counts were processed through Sysmex XS-1000i automatic hematology analyzers (Sysmax, Kobe, Japan) with supporting reagents. Coagulation indicators et al. were detected using Sysmex CS-5100 automatic coagulation analyzer (Sysmax, Kobe, Japan) was used for accessing coagulation indicators, which include APTT, TT, PT, D-Dimer, FDP, AT-IIII, and FVIII activity. Glucose-6-phosphate dehydrogenase (G6PD) enzyme activity was tested by using ELISA based quantitative assay. The biochemical indicators were tested on SIEMENS Advia 2400 Chemistry Analyzer (Deerfield, IL, USA).

### 2.3. Sanger Sequencing

QIAamp DNA Blood Mini Kit (Qiagen, Hilden, Germany) was used to extract the genomic DNA from peripheral blood following the manufacturer’s instructions. Complete F8 gene DNA covering all the coding and splice site regions was amplified using forward and reverse primers (Table 1) on the thermal cycler of ABI Model 9700 (Foster City, CA, USA), the resulted DNA products were processed using the ABI BigDye Terminator Cycle Sequencing Ready Reaction kit (Rotkreuz, Switzerland) and directly sequenced on the ABI Prism 3500 Genetic Analyzer. 

### 2.4. RNA and Reverse Transcription-PCR Analysis

Total RNA, extracted with Ambion TRIzol Reagent (Waltham, MA, USA), was reverse-transcribed (RT) using Superscript II kit (Life Technologies, Inc., Gaithersburg, MD, USA). Two specific F8 primers (Table 1) were used to identify and amplify the transcribed cDNA. The Amplified product is run on 2% agarose gels stained with ethidium bromide (5 mg/mL), and bands with expected sizes (709 bp for wild type patient, 709 bp and 144 bp for mutated patient) were separated by agarose gels. 

### 2.5. Ethics

The study was approved by ethics committee of Renmin Hospital of Wuhan University, and was conducted in compliance with the Declaration of Helsinki. The written informed consent was obtained from the parents.

### 2.6. Data Analysis

The sequenced F8 chromatograms were analyzed using NCBI Blast and UCSC genome blat programs and annotated according to GRCh38/hg38 reference assembly (NM_000132.4). F8 gene mutations was assigned based on the cDNA sequence (Gen-Bank No. NM_000132.4). The amino acid sequence of the mutated protein was determined by HGVS nomenclature (HGVS; http://www.hgvs.org/, 1 May 2022). The following databases and published data were referred to confirm the novelty of the mutation: dbSNP, clinvar, the Human Gene Mutation Database (HGMD; http://www.hgmd. cf.ac.uk/ac/, 10 May 2022), EAHAD Coagulation Factor Variant Databases (https://dbs.eahad.org/, 11 May 2022), EAHAD Coagulation Factor Variant Databases (EAHAD-CFDB; https://dbs.eahad.org/, 13 May 2022), Genome Aggregation Database (https://gnomad.broadinstitute.org/, 12 July 2022), 1000 Genomes (http://www.1000genomes.org/, 20 July 2022), and the Exome Variant Server (http://evs.gs.washington.edu/EVS/, (23 July 2022). Cross-species amino acid alignment was performed using ClustalW2 (http://www.ebi.ac.uk/Tools/msa/clustalw2/, 30 July 2022). The 3D structures of the wild-type and the mutant forms of C-terminal FVIII light chain were built with SWISS-Model by using the crystal structure pdb entry 7kwo.

## 3. Results

### 3.1. The Clinical Characteristics

A complete blood count, coagulation tests, glucose-6-phosphate dehydrogenase (G6PD) enzyme activity testing, and various biochemical indicators analyses were performed.

The complete blood count suggests that the proband had decreased red blood cell (RBC), platelet Count (PLT), and hemoglobin (Hb), indicating mild anemia. 

The G6PD enzyme activity is in the normal range, which can rule out g6pd deficiency. The coagulation function showed prolonged APTT, increased D-dimer and FDP, and decreased FVIII activity and Antithrombin III, suggesting moderate HA (Table 2). The RBC and Hb content of the other family members are in the normal range, except the proband’s mother and maternal grandmother had FVIII activity and coagulation function nearly at a critical threshold. 

### 3.2. F8 Gene Mutation Detected by Sanger Sequencing

**Figure 2:**
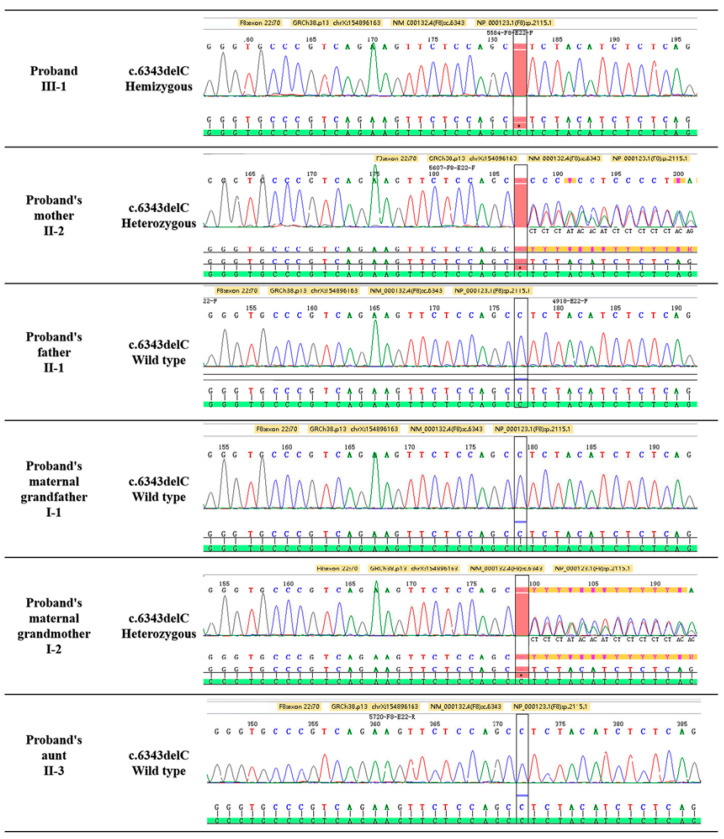
The F8 gene mutation detected in this family. The proband (III-1) carries the possible pathogenic hemizygous variant, the proband’s mother and maternal grandmother (II-2 and I-2) is a carrier of the variant. The other family members do not carry the mutation at this locus.

The Sanger sequencing identified a novel hemizygous variation F8 genetic variant c.6343delC (p.Leu2115SerfsTer28) in the proband (Figure 2). This variant generated owing to a deletion mutation in exon 22 of the F8 gene on the ChrX: 154,896,163 C locus, causing an exonic frame-shift mutation (c.6343delC, p.Leu2115SerfsTer28). This mutation was not reported from all public databases and our internal database. The pedigree analysis showed the inheritance model of the F8 genetic variant (Figure 1). The proband’s mother and maternal grandmother (Figure 1) was a carrier of the F8 genetic variant with a heterozygous genotype, the rest of the family members were wild-type. Only male members of the family are affected, indicating X-linked inheritance.

### 3.3. The F8 Mutation Is Pathogenic and Highly Conserved

**Figure 3:**
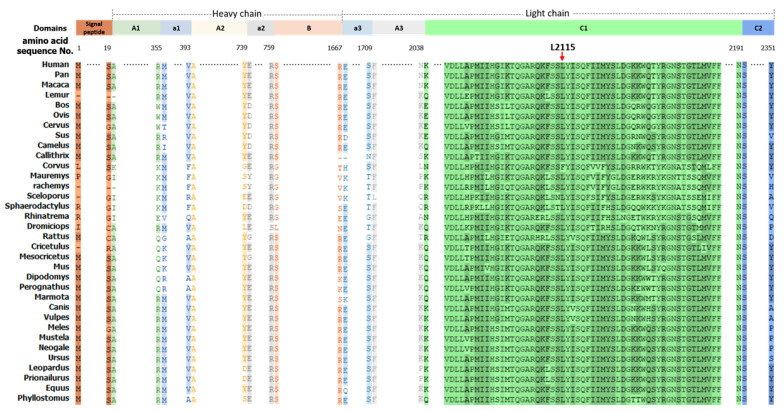
Multiple Sequence alignment of the F8 peptide sequences with affected residue (red frame) across 34 different species. The conservation of p.Leu2115 residue of F8 peptide sequences was examined across 34 different species by using the multiple-sequence alignment as shown in figure it note that the p.Leu2115 residue locates at C1 domain represented by red arrow, and is highly conserved across different species.

The conservation of c.6343delC (p.Leu2115SerfsTer28) residue of F8 was examined across 34 different species by using the publicly available multiple-sequence alignment, it was noted that the p.Leu2115 residue is highly conserved across mammalian species (Figure 3).

**Figure 4:**
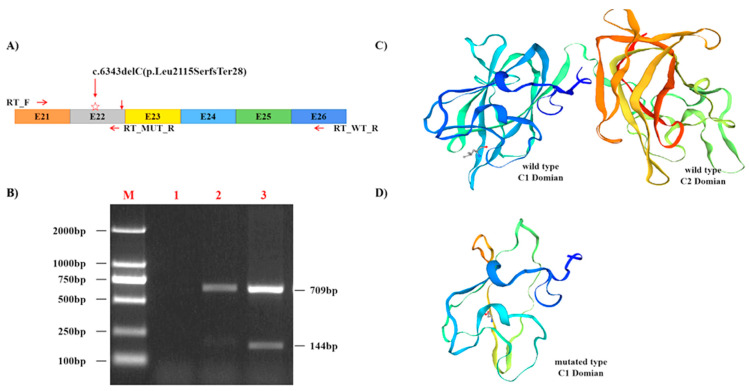
The cDNA amplification and crystal structure prediction of c.6343delC (p.Leu2115SerfsTer28) mutation. (**A**) Exon and domain structure of the F8 C-terminal encoding region. The NM_000132.4_c.6343delC (p.Leu2115SerfsTer28) mutation locating at exon 22 is indicated with a red down arrow and pentagram symbol. (**B**) RT-PCR was performed using primers located in exon 21 (RT_F) and exon 22 (RT_MUT_R and RT_WT_R) as shown. Two different products are present in the patient sample (lane 3). The upper band corresponds to the wild type product, the lower band corresponds to the product with deletion region from exon 23 to exon 26. (**C**,**D**) showed the wild-type (left) and mutant(right)structure of C-Terminal of human FVIII light chain (containing two FVIII domains: C1-C2) was predicted by Swiss PDB viewer using pdb entry 7kwo.1 taken from the Protein Data Bank. C showed the c.6343delC (p.Leu2115SerfsTer28) mutation of F8 lead to premature termination of translation and result in a truncated protein as a result of nonsense-mediated mRNA decay. The truncating mutation c.6343delC result in a truncated F8 protein from 2352 amino acids (6.95PI/267.05kDa, C) to 2142 amino acids (2.53PI/242.82kDa, D). The clustered residues under investigation (HGVS numbering) locating at C1 domain are represented by stick.

We applied SWISS-Model to compare the wild type (Figure 4A) and the mutant forms (Figure 4B) of C-terminal FVIII light chain by using the crystal structure pdb entry 7kwo.1 [8]. The p.Leu2115 residue is an exposed residue located in the C1 domain, shown as a stick, located in a random coil area of the FVIII structure (Figure 4). Our analysis showed that the exonic frame-shift mutation (p.Leu2115SerfsTer28) results in theoretical truncated proteins and interferes the secondary structures of the protein (Figure 4B), resulting in deletion of the C2 domain (Δ2193–2351) and a partial deletion of C1 domain (Δ2115–2192) (Figure 4C). 

## 4. Discussion

HA is a coagulation disorder characterized by partial or total deficiency of factor FVIII, caused by mutations in the gene encoding coagulation factor VIII. Up to now, in EAHAD F8 DATABASE v3.1 (https://dbs.eahad.org/, 13 May 2022), it has reported 3052 unique mutations corresponding to 10,144 individual HA patients. The predominant mutation types are point substitution (66.2%), deletions (23.4%), insertion (1.6%), fragment duplication (6%), and other types (2.8%) [9]. According to literature reports, F8 missense mutations usually caused non-severe HA, intron 22 inversion (45%), small deletions or insertions (16%), nonsense mutation (10%), large fragment deletion (3%), intron 1 inversion (2%), and splice site mutation (3%) [10,11,12], are always associated with severe HA. Moreover, more deleterious variations than neutral variations are observed in each of the A and C domains.

In this study, agarose gel electrophoresis and Sanger sequencing method were used for genetic diagnosis of a proband and family members with no family history of HA. A new pathogenic variant c.6343delC (p.Leu2115SerfsTer28) was detected in the 22th exon of the F8 gene (Figure 1). The American College of Medical Genetics and Genomics (ACMG) previously developed guidance are used for the interpretation of variants [13]. There were no records about c.6343delC mutation in the 1000G, EVS, ExAC, gnomAD, and HGMD databases prior to this study, which fulfilled criterion PM2 according to the ACMG guidelines. The F8 gene c.6343delC (p.Leu2115SerfsTer28) variant is frameshift mutation, which will result in truncated FVIII with total deletion of C2 domain and a partial deletion of C1 domain. It is reported that the C1/2 domains are essential for the binding of factor VIII to the von Willebrand factor and allowing formation of the intrinsic factor X complex [14], so the mutation lying within the C domain is likely to be causative. This evidence fulfil the mutation criterion PVS1 according to the ACMG guidelines. The FVIII activity and coagulation factor detection of the proband is consistent with HA, which met the criterion PP4 according to the ACMG guidelines. In summary, according to the ACMG’s criteria of pathogenicity, the F8 gene c.6343delC (p.Leu2115SerfsTer28) mutation is classified as pathogenic (PVS1 + PM2 + PP4) [13].

The proband’s mother and maternal grandmother was a heterozygous carrier, with normal thrombin time (TT) and FVIII activity at the edge of the Reference range, It has been reported that about 30% of F8 heterozygous women with factor VIII coagulation activity is less than 40% and have bleeding disorders (even if the affected family members are slightly involved) [15], carriers are more likely to bleed than unaffected women [5]. This phenomenon may be related to the "lyonization" of the X chromosome [16]. It is well-established that avoiding the marriage of close relatives and carrying out pre-pregnancy screening and prenatal diagnosis for female carriers of HA are fundamental measures to reduce the birth rate of children with hemophilia, which is of great significance for improving the quality of the population and reducing the burden on the family and society. The sequence analysis of F8 gene is of great significance for carrier screening and disease diagnosis, and has important guiding significance for gene therapy of HA.

Prophylaxis with FVIII replacement is the standard of care in hemophilia A, for achieving a level of hemostasis control that allows patients to meet their lifestyle goals [17]. It has been proved that a smaller dose of factor VIII concentrate may keep the affected children a higher than 1% FVIII activity, which can play a preventive role [18,19]. However, the frequently intravenous infusions and the potential immune response to the infusion concentrates, resulting in the production of inhibitory antibodies, making patients vulnerable to illness and disability. 

It has been reported that the types and location of F8 gene mutations are related with the risk of adverse immune reaction to replacement therapy, some types of F8 LOF (loss-of-function) variants, such as frameshifts, inversions breaking, gross deletions, etc., had the highest risk for inhibitor development. Interestingly, nonsense mutations at the 5′ regions of F8 reflect a lower risk than those at the 3′ regions, which was explained by the possibility of several internal initiations of mRNA translation excellent matching to the Kozak consensus. The 5′ mutations may have a more possibility to translate part of the F8 coding sequence located 3′ to the nonsense mutation, the FVIII-related polypeptides favor immune tolerance to therapeutic FVIII [20]. In view of the fact that the patient carries a frameshift mutation located at the 3′ end, our genetic results may mean that the patient in this family need a smaller dose of factor VIII concentrate to keep a normal function, and at the same time, there is an unignorable risk of producing inhibitors in the future.

At present, recombinant products with a relatively long half-life and complex drugs using a combination of multiple activated coagulation factors have been developed to improve the efficacy of alternative therapies. Gene therapy based on adeno-associated virus (AAV) vectors and gene editing technology is an emerging strategy for the treatment of hemophilia, and has been successfully applied to animal models [21,22,23,24].

## 5. Conclusions

In this study, a novel frameshift deletion mutation in the F8 gene was identified for the first time in a Chinese HA family by using co-segregation analysis and Sanger sequencing. This study contributed a new mutation to the F8 gene pathogenic mutation spectrum, and clarified its pathogenicity, patient’s blood coagulation function, and clinical manifestations. The direct mutation analysis or indirect linkage analysis of HA has become a feasible option for couples with high risk of having an affected child, genetic counseling about the accuracy and limitations of tests should be provided for couples who request preimplantation genetic diagnosis (PGD).

## Figures and Tables

**Table 1:** Primer pairs sequence and PCR conditions for F8 amplification.

Primer Name	Primer Sequence	Tm Value (°C)	GC Content (%)	Product Size (bp)
**E1**	F: 5′-GGGTAAAGTTCCTTAAAATGCTCT-3′ R: 5′-TATGACGAAGAGAAGCAATGGACA-3′	57 60	37.5 41.67	536
**E2**	F: 5′-AAAGGATTTTTGGCTGATGCAGT-3′ R: 5′-TGGAGGTAAGCAGTTGGCATAG-3′	60 60	39.13 50	572
**E3**	F: 5′-ACTGCACCTCCAGTGCATAAG-3′ R: 5′-GTAACGCCACCATTACAAAGCA-3′	60 60	52.38 45.45	467
**E4**	F: 5′-ATGCAGAAAGTCCGTTTCTTATGT-3′ R: 5′-TCAGGTGAAGGAACACAAATGC-3′	59 59	37.5 45.45	422
**E5**	F: 5′-GAGACCTGACATCAAAGCCAAG-3′ R: 5′-TGAGGCCAGTCCGTTATCCA-3′	59 61	50 55	435
**E6**	F: 5′-F: 5′-TGAGAGGATAAGTGCTGTGTGC-3′ R: 5′-GATGCCGAGCTGTTTGTGAACT-3′	60 62	50 50	348
**E7**	F: 5′-ACGAATGAATGGTCAACAGGTGC-3′ R: 5′-GAAGCTGGAAACTAGGGGATCT-3′	62 59	47.83 50	628
**E8**	F: 5′-GGAAGGCCTAATAAGAGAGAAAGAA-3′ R: 5′-ATAGTCCCAGTCCTCCTCTTCA-3′	58 59	40 50	686
**E9**	F: 5′-CAAACCAACAAATCCTGAAGCCA-3′ R: 5′-GTGTCGAGTTTAGTGGGTGACATTA-3′	60 61	43.48 44	598
**E10**	F: 5′-GACCACAGTTTTCTTGTTGATCCT-3′ R: 5′-TGTCAGGCGACTCTTCACGA-3′	59 61	41.67 55	358
**E11**	F: 5′-AATAGGTGCGACTTTAGCTTCCA-3′ R: 5′-TCCATCCAGCAGGCACGTTT-3′	60 62	43.48 55	517
**E12**	F: 5′-GGATCAGTCACCCTCTTGTCC-3′ R: 5′-GGGTTATATGATCACGTGTGTTTGA-3′	60 59	57.14 40	545
**E13**	F: 5′-CCTGGGAATAAGATAATGGGCA-3′ R: 5′-ATCCCTGTACCTCAAGGAAGAAAA-3′	58 59	45.45 41.67	451
**E14_1**	F: 5′-AGGCATAGTACAACAGCAGCAA-3′ R: 5′-ATGAACTGGCATACTTGGGGG-3′	60 60	45.45 52.38	873
**E14_2**	F: 5′-TCCATCAGACAATTTGGCAGCAG-3′ R: 5′-GAGGCAAAACTACATTCTCTTGGA-3′	62 59	47.83 41.67	971
**E14_3**	F: 5′-CCAAGCAGCAGAAACCTATTTCTTA-3′ R: 5′-TTGGGCAAGTCTGGTTTCGG-3′	60 61	40.00 55.00	956
**E14_4**	F: 5′-AAGCAGTCATTTCTTACAAGGAGC-3′ R: 5′-TCATTGTTGGTGTCATCATCTGGTA-3′	60 60	41.67 40.00	948
**E15**	F: 5′-CAAAATGCTTCTCAGGCACCTA-3′ R: 5′-ATGTGCAAGGGACATTACCAA-3′	59 58	45.45 42.86	731
**E16**	F: 5′-TCTGTACCACTTCTTCCAGGGT-3′ R: 5′-CCATCCTCTTCAGTAGATTCCAGA-3′	60 59	50 45.83	524
**E17_E18**	F: 5′-TGGAATCTACTGAAGAGGATGGATT-3′ R: 5′-CACTGATTGTGTTCCCAGTGC-3′	59 60	40 52.38	847
**E19**	F: 5′-CCCCCAACTGTAAGGGTCAC-3′ R: 5′-CCTGACACAAGCAACCATTCC-3′	60 60	60 52.38	374
**E20**	F: 5′-GCATTTGTTGACGTTCTCCCAT-3′ R: 5′-GGAGAGGAGGAGATGTATTTGAGAGG-3′	60 62	45.45 50	314
**E21**	F: 5′-TGTTTTTCTCTATTTTCACCACAGC-3′ R: 5′-CCCCATATCTCTTTGTTCATGACTG-3′	59 59	36 44	364
**E22**	F: 5′-GGTGACTGCTTCACTTGCACA-3′ R: 5′-GAGCCTTGACACTACTACATTTTTG-3′	61 58	52.38 40	475
**E23**	F: 5′-CTTCACTTGCCCCAGACCTAAT-3′ R: 5′-CCCAGGACTATGCTGGTTTTAGC-3′	60 61	50 52.17	512
**E24**	F: 5’-TGCAAAAGTTAAAACCTGAGAAATG-3′ R: 5’-GTCTGCCCATAACCAAACTTCC-3′	57 60	32 50	422
**E25**	F: 5′-AGAGTGAGAAGTGCTGTGGTATGG-3′ R: 5′-AAAGTCACTGTGTTCTCTCAGAATG-3′	62 59	50 40	446
**E26**	F: 5′-TCCCAGATGCGTAGGACAGAGT-3′ R: 5′-AGCACAAAGGTAGAAGGCAAGC-3′	63 61	54.55 50	398
**RT_MUT**	F: 5′-TGGATCAAGGTGGATCTGTTGG-3′ R: 5′-TCCTCGATAAGTCTGCCACT-3′	58 56	50.00 50.00	144
**RT_WT**	F: 5′-TGGATCAAGGTGGATCTGTTGG-3′ R: 5′-GGTAGCGAGTCAGTAACGGTG-3′	58 58	50.00 57.14	709

Note: F, forward; R, reverse; Tm, melting temperature.

**Table 2:** The clinical laboratory tests show coagulation evaluations of the proband and his family.

Test Items	Proband	Mother	Father	Aunt ^#^	Grandmother ^#^	Reference Ranges
**RBC (10^12^ cells/L)**	2.43↓	4.28	4.52	4.50	3.70	3.6–6.6 (Children) 4.3–5.8 (Male) 3.8–5.1 (Female)
**Hb (g/L)**	86↓	115	139	133	113	140–200 (Children) 130–175 (Male) 115–150 (Female)
**PLT (10^9^ cells/L)**	185↓	209	168	268	307	242–378 (Children) 125–350 (Adult)
**PT (s)**	11.20	12.50	10.80	11.30	10.40	9–13
**APTT (s)**	>170↑	36.80	28.7	29	37.80	20–40
**TT (s)**	15.40	16.30	17.30	14.30	15.90	14–21
**D-dimer (mg/L)**	6.26↑	0.51	0.18	0.32	0.48	0–0.55
**FDP (mg/L)**	15.26↑	1.80	1.50	2.07	2.26	0–5
**VWF** **:Ag**	61.90	65.30	103.20	71.80	123.50	50–200
**AT-III (%)**	44.80↓	85.30	94.80	82.70	94.40	80–120
**FVIII activity (%)**	2.0↓	75.40	154.30	116.50	69.70	60–189
**FIX activity (%)**	38.90↓	100.70	95.00	120.10	74.40	65–150
**FX activity (%)**	74.0↓	93.30	97.40	90.30	90.10	77–131
**FXI activity (%)**	67.60	100.70	71.70	101.70	81.20	65–150
**FXII activity (%)**	19.30↓	63.50	71.40	86.70	69.90	50–150
**G6PD enzyme activity (U/L)**	3491.4	3193.4	2954.3	2485.4	2103.5	2500–5800 (Newborn) 1700–4000 (Children) 1300–3600 (Adult)

Note: RBC, red blood cell count; Hb, hemoglobin concentration; PLT, platelet count; PT, Prothrombin Time; APTT, Activated Partial Thromboplastin Time; TT, Thrombin Time; FDP, fibrin degradation product; AT, III-Antithrombin III. ^#^ Maternal, ↑ increased, ↓ decreased.

## Data Availability

The data that support the findings of this study are available on request from the corresponding author. The data are not publicly available due to privacy or ethical restrictions.

## References

[1] Soucie J.M., Miller C.H., Dupervil B., Le B., Buckner T.W. (2020). Occurrence rates of haemophilia among males in the United States based on surveillance conducted in specialized haemophilia treatment centres. Haemoph. Off. J. World Fed. Hemoph..

[2] Dai J., Lu Y., Ding Q., Wang H., Xi X., Wang X. (2012). The status of carrier and prenatal diagnosis of haemophilia in China. Haemoph. Off. J. World Fed. Hemoph..

[3] Chistolini A., Papacchini M., Mazzucconi M.G., La Verde G., Arcieri R., Ferrari A., Paesano R., Pachi A., Mariani G. (1990). Carrier detection and prenatal diagnosis in haemophilia A and B. Haematologica.

[4] White G.C., Rosendaal F., Aledort L.M., Lusher J.M., Rothschild C., Ingerslev J., Factor V., Factor I.X.S. (2001). Definitions in hemophilia. Recommendation of the scientific subcommittee on factor VIII and factor IX of the scientific and standardization committee of the International Society on Thrombosis and Haemostasis. Thromb. Haemost..

[5] Paroskie A., Gailani D., DeBaun M.R., Sidonio R.F. (2015). A cross-sectional study of bleeding phenotype in haemophilia A carriers. Br. J. Haematol..

[6] Stoilova-McPhie S., Lynch G.C., Ludtke S., Pettitt B.M. (2013). Domain organization of membrane-bound factor VIII. Biopolymers.

[7] Dai J., Wang X. (2020). The current situation of genetic diagnosis of hemophilia. J. Clin. Intern. Med..

[8] Fuller J.R., Knockenhauer K.E., Leksa N.C., Peters R.T., Batchelor J.D. (2021). Molecular determinants of the factor VIII/von Willebrand factor complex revealed by BIVV001 cryo-electron microscopy. Blood.

[9] McVey J.H., Rallapalli P.M., Kemball-Cook G., Hampshire D.J., Giansily-Blaizot M., Gomez K., Perkins S.J., Ludlam C.A. (2020). The European Association for Haemophilia and Allied Disorders (EAHAD) Coagulation Factor Variant Databases: Important resources for haemostasis clinicians and researchers. Haemoph. Off. J. World Fed. Hemoph..

[10] Gouw S.C., van den Berg H.M., Oldenburg J., Astermark J., de Groot P.G., Margaglione M., Thompson A.R., van Heerde W., Boekhorst J., Miller C.H. (2012). F8 gene mutation type and inhibitor development in patients with severe hemophilia A: Systematic review and meta-analysis. Blood.

[11] Marder V.J., Aird W.C., Bennett J.S., Schulman S., White G.C. (2012). Hemostasis and Thrombosis: Basic Principles and Clinical Practice.

[12] Kaufman R.J., Powell J.S. (2013). Molecular approaches for improved clotting factors for hemophilia. Hematol. Am. Soc. Hematol. Educ. Program..

[13] Richards S., Aziz N., Bale S., Bick D., Das S., Gastier-Foster J., Grody W.W., Hegde M., Lyon E., Spector E. (2015). Standards and Guidelines for the Interpretation of Sequence Variants: A Joint Consensus Recommendation of the American College of Medical Genetics and Genomics and the Association for Molecular Pathology. Genet. Med. Off. J. Am. Coll. Med. Genet..

[14] Liu Z., Lin L., Yuan C., Nicolaes G.A., Chen L., Meehan E.J., Furie B., Furie B., Huang M. (2010). Trp2313-His2315 of factor VIII C2 domain is involved in membrane binding: Structure of a complex between the C2 domain and an inhibitor of membrane binding. J. Biol. Chem..

[15] Plug I., Mauser-Bunschoten E.P., Bröcker-Vriends A.H.J.T., van Amstel H.K.P., van der Bom J.G., van Diemen-Homan J.E.M., Willemse J., Rosendaal F.R. (2006). Bleeding in carriers of hemophilia. Blood.

[16] Favier R., Lavergne J.-M., Costa J.-M., Caron C., Mazurier C., Viémont M., Delpech M., Valleix S. (2000). Unbalanced X-chromosome inactivation with a novel FVIII gene mutation resulting in severe hemophilia A in a female. Blood.

[17] Aledort L., Mannucci P.M., Schramm W., Tarantino M. (2019). Factor VIII replacement is still the standard of care in haemophilia A. Blood Transfus..

[18] Fischer K., Astermark J., van der Bom J.G., Ljung R., Berntorp E., Grobbee D.E., van den Berg H.M. (2002). Prophylactic treatment for severe haemophilia: Comparison of an intermediate-dose to a high-dose regimen. Haemoph. Off. J. World Fed. Hemoph..

[19] Feldman B.M., Pai M., Rivard G.E., Israels S., Poon M.C., Demers C., Robinson S., Luke K.H., Wu J.K.M., Gill K. (2006). Tailored prophylaxis in severe hemophilia A: Interim results from the first 5 years of the Canadian Hemophilia Primary Prophylaxis Study. J. Thromb. Haemost..

[20] Green P.M., Bagnall R.D., Waseem N.H., Giannelli F. (2008). Haemophilia A mutations in the UK: Results of screening one-third of the population. Br. J. Haematol..

[21] Chen H., Shi M., Gilam A., Zheng Q., Zhang Y., Afrikanova I., Li J., Gluzman Z., Jiang R., Kong L.-J. (2019). Hemophilia A ameliorated in mice by CRISPR-based in vivo genome editing of human Factor VIII. Sci. Rep..

[22] Li X., Zhang J., Zhang L., Cheng T., Zhang X. (2015). Research advances on gene therapy for hemophilia A. Zhonghua Xue Ye Xue Za Zhi.

[23] Sung J.J., Park C.-Y., Leem J.W., Cho M.S., Kim D.-W. (2019). Restoration of FVIII expression by targeted gene insertion in the FVIII locus in hemophilia A patient-derived iPSCs. Exp. Mol. Med..

[24] Hu Z., Zhou M., Wu Y., Li Z., Liu X., Wu L., Liang D. (2019). ssODN-Mediated In-Frame Deletion with CRISPR/Cas9 Restores FVIII Function in Hemophilia A-Patient-Derived iPSCs and ECs. Mol. Ther. Nucleic Acids.

